# Special Issue: Monoclonal Antibodies

**DOI:** 10.3390/antib7020017

**Published:** 2018-04-10

**Authors:** Christian Klein

**Affiliations:** Roche Pharmaceutical Research & Early Development, 8952 Schlieren, Switzerland; christian.klein.ck1@roche.com

Monoclonal antibodies are utilized in clinical practice for the treatment of various diseases including cancer, autoimmunity, metabolic and infectious diseases. Over the last 20 years monoclonal antibodies have established themselves as therapeutics and various so-called “blockbuster” drugs are in fact antibodies. Currently ca. 80 monoclonal antibodies have been approved in Europe and the US and several hundreds of them are currently in early and advanced clinical trials. Notably, the field has significantly advanced in the last decade, and nowadays, a large proportion of antibodies in development are engineered antibodies including bispecific antibodies, antibody drug conjugates and novel antibody-like scaffolds. This Special Issue on “Monoclonal Antibodies” includes original manuscripts and reviews covering various aspects related to the discovery, analytical characterization, manufacturing and development of therapeutic and engineered antibodies.

The collection starts with a number of reviews. Cho and colleagues [1] review the state of the art in the therapy of multiple myeloma where antibody-based immunotherapies are changing the current treatment paradigm, and Wang-Lin and Balthasar summarize pharmacokinetic and pharmacodynamic considerations that are important for the treatment of bacterial infections by monoclonal antibodies [2]. Finally, Fülöp and colleagues review the role of complement activation in infusion reactions associated with the application of monoclonal antibodies and the potential use of complement factor H for its prevention [3].

A series of original articles describes novel monoclonal antibodies for potential diagnostic or therapeutic application. Rashidian and colleagues describe a novel rabbit monoclonal antibody MRQ-67 that specifically recognize the R132H mutation of isocitrate dehydrogenase 1 (IDH1) which are prevalent in diffuse astrocytomas, oligodendrogliomas, and secondary glioblastomas but not the wildtype IDH1. MRQ-67 is able to identify neoplastic cells in glioma tissue specimens and can be used as a tool in glioma subtyping [4]. Zhang and colleagues have identified novel monoclonal antibodies against the Plasmodium falciparum circumsporozoite protein that is a major and immunodominant protective antigen on the surface of plasmodium sporozoites [5]. These antibodies are specific for the central repeat region and mediate protection against challenges from sporozoites. Finally, Rocha and colleagues generated antibodies directed against novel epitopes of the Dengue nonstructural protein 1 (NS1) which is a multi-functional glycoprotein essential for viral replication and modulation of host innate immune responses and represents a surrogate marker for infection [6]. These antibodies are able to differentiate Dengue and Zika virus infections and may contribute to the development of novel diagnostic tools.

In a series of three articles, Strube and colleagues [7,8,9] describe approaches useful for the manufacturing and analytical characterization of monoclonal antibodies. An article by Schmidt et al. [7] describes aqueous two-phase extraction (ATPE) as a method to capture monoclonal antibodies using a combined harvest and capture step during the downstream process. A subsequent article by Kornecki et al. focuses on the characterization and classification of host cell proteins (HCPs) and how to categorize and avoid them in the manufacturing process [8]. Finally, Zobel-Roos et al. [9] propose a process analytical approach allowing for controlled automation of the downstream process by inline concentration measurements based on UV/VIS spectral analysis. In the same area, Radhakrishnan and colleagues show how time-dependent media supplementation by MnCl2 can be used to control the glycosylation profile of antibodies [10]. Castellanos and colleagues use small-angle scattering (SAS) combined with size-exclusion multi-angle light scattering high-performance liquid chromatography and molecular modeling to characterize antibody-antigen complexes in solution [11].

Lastly, two articles deal with engineering monoclonal and bispecific antibodies. Tam and colleagues [12] have identified a set of novel mutations in the Fc-portion of antibodies that abrogate the immune effector function of the respective antibodies. Such Fc-mutations are essential for the development of antibody therapeutics where simultaneous FcgR activation is undesired for the mechanism of action, e.g., for T-cell bispecific antibodies. Dheilly and colleagues [13] engineered novel CD47-CD19 bispecific antibodies based on low affinity CD47 inhibitory antibodies. The corresponding CD47-CD19 bispecific antibody inhibited tumor growth in vivo and induced a long lasting anti-tumor immune response that could be further enhanced in combination with chemotherapy or PD-1/PD-L1 checkpoint blockade.

This collection of articles should be of value to readers working in the field of monoclonal and therapeutic antibodies.
